# Altered Morpho-Functional Features of Neurogenesis in Zebrafish Embryos Exposed to Non-Combustion-Derived Magnetite

**DOI:** 10.3390/ijms25126459

**Published:** 2024-06-12

**Authors:** Pietro Cacialli, Serena Ricci, Giulia Pia Servetto, Valeria Franceschini, Francisco Ruiz-Zepeda, Ruggero Vigliaturo

**Affiliations:** 1 Department of Biological, Geological and Environmental Sciences (BIGEA), University of Bologna, 40126 Bologna, Italy; 2 Department of Earth Sciences, University of Turin, 10124 Turin, Italy; 3 Department of Physics and Chemistry of Materials, Institute of Metals and Technology, Lepi pot 11, 1000 Ljubljana, Slovenia; 4 Department of Materials Chemistry, National Institute of Chemistry, Hajdrihova 19, 1000 Ljubljana, Slovenia; 5 Interdepartmental Centre for Studies on Asbestos and Other Toxic Particulates “G. Scansetti”, University of Turin, 10124 Turin, Italy

**Keywords:** zebrafish, neurogenesis, microglia, non-combustion-derived magnetite, oxidative stress

## Abstract

Neurogenesis is the process by which new brain cells are formed. This crucial event emerges during embryonic life and proceeds in adulthood, and it could be influenced by environmental pollution. Non-combustion-derived magnetite represents a portion of the coarse particulate matter (PM) contributing to air and water pollution in urban settings. Studies on humans have reported that magnetite and other iron oxides have significant damaging effects at a central level, where these particles accumulate and promote oxidative stress. Similarly, magnetite nanoparticles can cross the placenta and damage the embryo brain during development, but the impact on neurogenesis is still unknown. Furthermore, an abnormal Fe cation concentration in cells and tissues might promote reactive oxygen species (ROS) generation and has been associated with multiple neurodegenerative conditions. In the present study, we used zebrafish as an in vivo system to analyze the specific effects of magnetite on embryonic neurogenesis. First, we characterized magnetite using mineralogical and spectroscopic analyses. Embryos treated with magnetite at sub-lethal concentrations showed a dose–response increase in ROS in the brain, which was accompanied by a massive decrease in antioxidant genes (*sod2*, *cat*, *gsr*, and *nrf2*). In addition, a higher number of apoptotic cells was observed in embryos treated with magnetite. Next, interestingly, embryos exposed to magnetite displayed a decrease in neural staminal progenitors (*nestin*, *sox2*, and *pcna* markers) and a neuronal marker (*elavl3*). Finally, we observed significative increases in *apoeb* (specific microglia marker) and interleukin-1b (*il1b*), confirming a status of inflammation in the brain embryos treated with magnetite. Our study represents the very first in vivo evidence concerning the effects of magnetite on brain development.

## 1. Introduction

Neurogenesis represents a complex process, which occurs during embryogenesis and throughout a person’s lifetime, through which neural stem cells can originate multiple mature cell types—neurons, astrocytes, and oligodendrocytes [1]. During development, once the neural tube is formed within the lumen, and it is possible to observe a high number of asymmetrically dividing neuronal progenitors, which express molecular markers of stemness. The *neurog1* (neurogenin1) gene is one of the first pro-neural genes to produce transcription factors together with *asc1* (achaete-scute1) [2,3]. These transcription factors are known as the *basic Helix-Loop-Helix (bHLH)*. Under the strict spatio-temporal control of “neurogenetic gradients”, progenitors give rise to the formation of differentiated cells. *Shh* has a key signal inducer role and is needed for the concentration-dependent activation of *neurog1* and *asc1*, which causes the formation of thalamic nuclei in the vertebrates [4]. The balance between the differentiation and self-renewal of progenitors is related to the downregulation of stemness markers and the upregulation of specificity markers [5,6]. All neurogenetic processes are perfectly orchestrated from several regulating pathways, which act in different brain regions and involve multiple factors, including genetic, physiologic, and environmental ones [7,8]. Pollution represents one of the major environmental factors impacting brain physiology, both in adulthood and during development [9]. Exposure to metal-bearing particulate matter (PM), indeed, has been widely associated with neurodevelopmental and neurodegenerative disorders and cognitive dysfunctions [10,11]. Both fine (PM2.5) and coarse (PM10) particles can enter the body through the respiratory and gastrointestinal tracts, so they can gain access to multiple organs, including the brain, depending on their physical and chemical properties (e.g., morphometry or surface charge) [12]. While the European Union’s zero-pollution action plan is successfully reducing the number of premature deaths caused by PM2.5 [13], association studies across different countries/regions have highlighted an increased risk in daily all-cause mortality due to coarse PM inhalation [14]. Magnetite (Fe^2+^Fe^3+^_2_O_4_) represents the main PM10 component of the underground transportation system. In addition, in urbanized areas, the water distribution system can act as an effective sink for atmospheric PM [15,16]. Fe accumulation in organisms is associated with increased reactive oxygen species (ROS) production and oxidative stress driven by Fenton reactions, which can induce neurotoxicity [17]. Indeed, several neurodegenerative diseases such as Parkinson’s disease (PD) and Alzheimer’s disease (AD) are associated with abnormal concentrations of iron [18]. Studies on humans, as highlighted by Gieré, magnetite nanoparticles and other nanosized Fe oxides can easily enter through the olfactory system [19], cross the blood–brain barrier, and reach the brain tissues, contributing to oxidative-stress-based brain damage [20]. The toxic effects of magnetite on neuronal function seem to be related to magnetite’s ability to trigger oxidative stress and promote neuronal inflammation [21]. In particular, several studies have shown that the presence of magnetite nanoparticles in brain samples from AD subjects is strictly associated with beta-amyloid plaques [18,22,23]. A similar association has been reported in brain-degenerative features associated with PD [24]. In addition, in vitro studies have suggested that iron oxide nanoparticles, including magnetite, can cross the placental barrier, reaching the embryo and inevitably affecting its development [25,26]. One study performed in chicken revealed that maternal exposure to magnetite allowed these particles to reach the embryo, causing damaging cerebral effects [27]. Nevertheless, at the present time, no evidence has shown whether cleavage fragments detaching from coarse magnetite particles or elemental Fe released by coarse magnetite particles can affect brain cell formation processes during development. One in vitro study on mouse pluripotent stem cells showed that immoderate magnetite stimulation can impact neuronal differentiation and can affect the function of more susceptible neurons, such as dopaminergic ones [28]. Nevertheless, no similar report in vivo is available. In the present study, therefore, we aim to investigate the potential toxicity of non-combustion-derived magnetite particles on embryonic brain cell formation by using one of the most advantageous animal models in the field of neurobiology research: zebrafish (*Danio rerio*) [29,30]. The zebrafish is a teleost fish, belonging to the family of *Cyprinidae*, presenting different characteristics (e.g. small size, high fecundity, ex utero and transparent offspring, rapidity of embryonic development, etc.) that make it a formidable animal model for conducting toxicology studies related to both embryogenesis and adulthood [31]. In the last 20 years, this small fish also became an excellent model for the in vivo investigation of neural regeneration and neurogenesis, as it has nervous system cellular physiology that is sufficiently analogous to that of mammals [32,33]. Here, we firstly characterized magnetite nanoparticles by mineralogical and spectroscopic analyses, and then we exposed zebrafish larvae at different sub-lethal concentrations of magnetite to analyze oxidative stress patterns and the expression of marker genes of neuronal precursors, as well as of more differentiated brain cells.

## 2. Results

### 2.1. Mineralogical Characterization of Magnetite

The characterization of commercial magnetite was performed to confirm the commercial sample mineralogical identity and the semi-quantitative chemical composition. The characterization of commercial samples is routinely performed by our laboratory for each use of any inorganic compounds to assure the repeatability of the experiments, since variation between different batches of the same product might occur.

**Figure 1:**
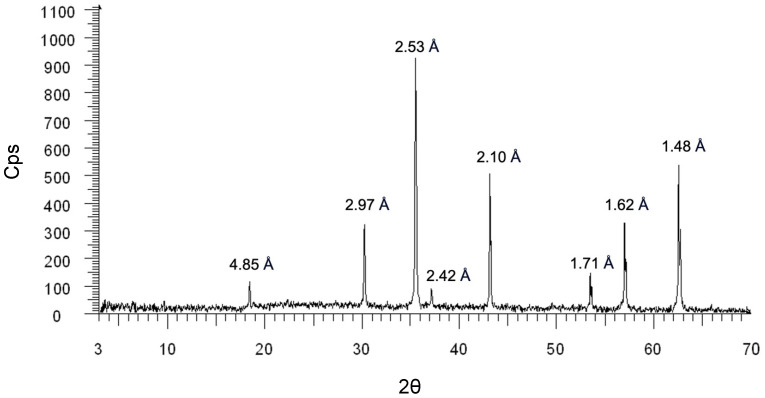
PXRD spectrum collected on the non-combustion-derived magnetite sample. The significative d_hkl_ spacings are indicated above each peak and are inversely proportional to the detected 2θ values indicated in the *x*-axis. Counts per second are indicated as “Cps”.

The PXRD spectrum and the peaks within match with the expected ones for magnetite crystals (JCPDS record 00-019-0629) described in the literature (Figure 1). The SEM-EDXS spot semi-quantitative standardless chemical analyses confirmed the presence of only Fe-based mineral phases.

### 2.2. Morphometric Evaluation of Magnetite

Next, we performed a morphometric evaluation of commercial magnetite particles by means of SEM secondary electron imaging.

**Figure 2:**
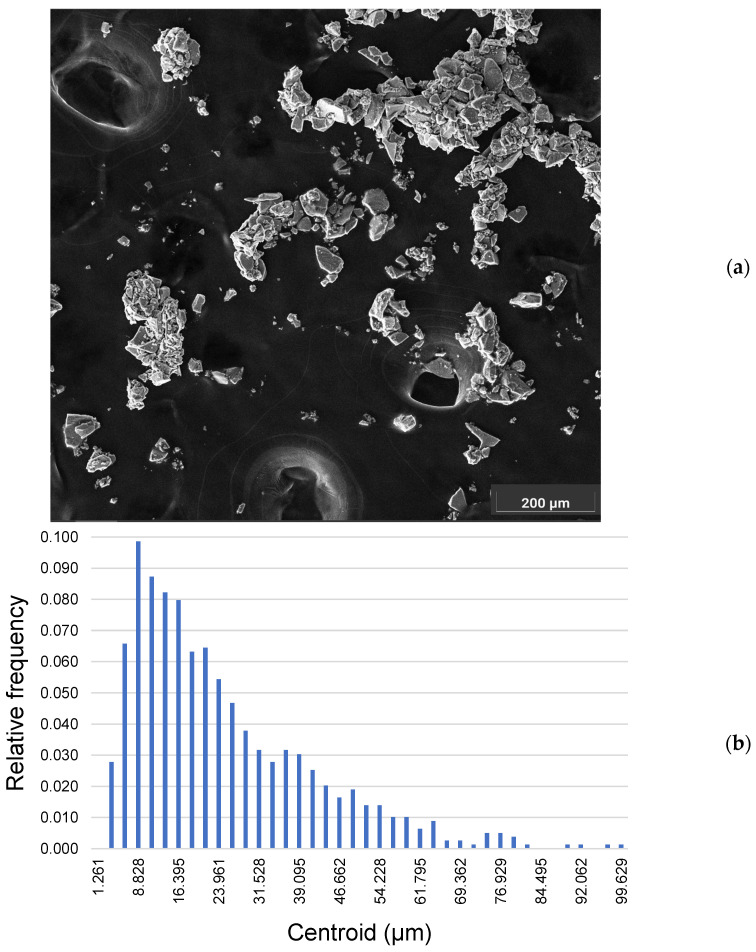
(**a**) Magnetite particles and agglomerates in a secondary electron image acquired by SEM at 1000× magnification; (**b**) dimensional distribution of the Feret diameters (SEM data). The relative frequency of the number of particles (*n* = 791) distributed in each bin is indicated on the *y*-axis, while the centroids of each bin in which the particles of the related dimensional range are assigned are indicated on the *x*-axis.

The particles have a homogeneous massive morphology, with smooth facets and well-defined edges (Figure 2a). Some of the particles are collected in aggregates. These aggregates achieve a dimension of over 200 µm. The presence of aggregates is a consequence of the ethanol droplet drying when transferred onto the carbon tape. The dimensional distribution of the particles’ Feret diameter has a Gaussian right skewed shape (Figure 2b). The dimensional distribution has a range that goes from a Feret diameter of 2.904 µm to 99.890 µm (mean Feret diameter 24.702 µm; σ_(*n*−1)_ = 16.979; *n* = 791). Most of the particles in this distribution are assigned to the bin that has a centroid located at 8.828 µm (having a minimum at 7.570 µm and a maximum at 10.090 µm), while the mean diameter is 24.702 µm. Following the bin with the higher number of particles (centroid located at 8.828 µm), the more populated bins were the ones with the centroids at 11.350 µm, 13.872, and 16.395 µm.

### 2.3. Atomic Resolution Microscopy and Dual-EELS on Magnetite Nanoparticles and Cleavage Fragments

**Figure 3:**
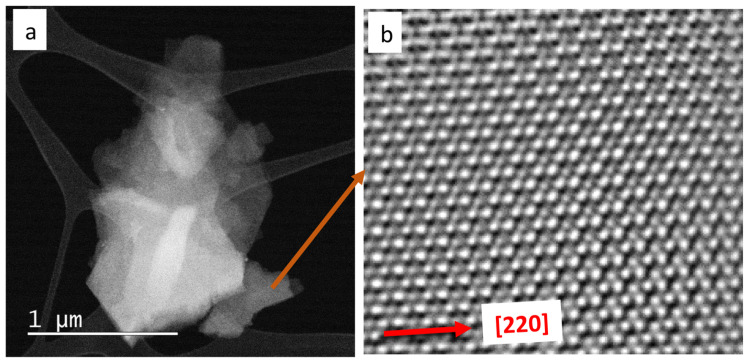
(**a**) Agglomerate of magnetite nanoparticles or cleavage fragments observed in Medium-Angle Annular Dark Field (MAADF); (**b**) atomic resolution detail of a region of interest (orange arrow origin). The image was filtered using an Inverse Fast Fourier Transform (IFFT) function to improve the visibility. Red arrow indicates the crystallographic direction of the ROI.

The presence of a small amount of magnetite nanoparticles or cleavage fragments in the particle population given to the zebrafish larvae was confirmed by ARM observations (Figure 3a). All nanoparticles and cleavage fragments were crystalline and compatible with the magnetite crystal lattice (Figure 3b). The EDXS signal related to Si-based compounds was detected in some of the observed particles. The Dual-EELS analyses allowed us to determine an average valence state of 2.48 (σ*_n_*_−1_ = 0.11) at the boundary of the magnetite particles (*n* = 15).

### 2.4. Toxicological Monitoring of Zebrafish Embryos

To define sub-lethal concentrations of magnetite (M), at 2 hpf 180 embryos (30 for each condition) were collected and treated with water-suspended magnetite at different concentrations (0; 100; 200; 400; 600; 800 µg/mL).

**Figure 4:**
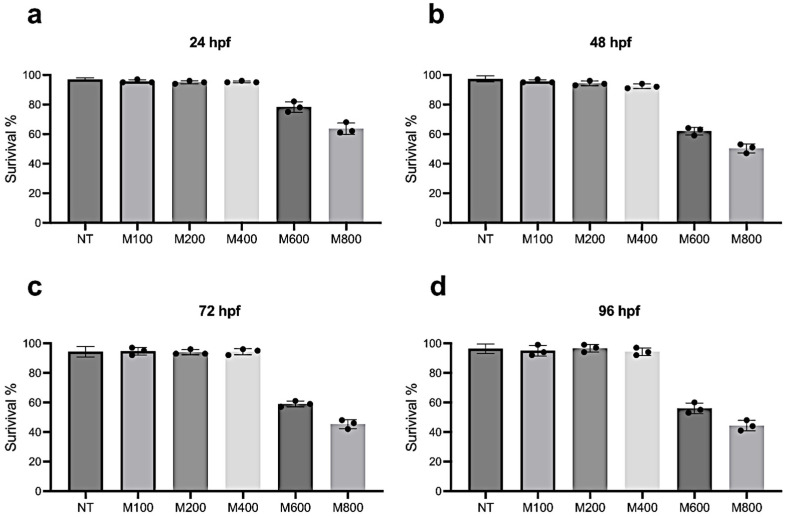
Percentage of survival of non-treated zebrafish embryos and those treated with magnetite (M) at different concentrations at 24 hpf (**a**); 48 hpf (**b**); 72 hpf (**c**); and 96 hpf (**d**).

Survival was monitored every 24 h. The survival rate was described as the percentage of dead fishes after 96 h as compared with the control group. As shown in the figure (Figure 4a–d), we observed a significant decrease in survival of the embryos treated with high concentrations of magnetite (600 and 800 µg/mL). A morphological evaluation of the dead embryos underlined the presence of multiple tissue defects, such as heart edema and impaired blood circulation coat or tail damaged (Supplementary Figure S1). Differently, no significant differences in survival rates and morphology were observed among the groups M100, M200, and M400. Thus, we decided to use these sub-lethal concentrations for the further experiments.

### 2.5. Magnetite Exposure Increases Oxidative Stress and Apoptosis in Zebrafish Embryo Brain

**Figure 5:**
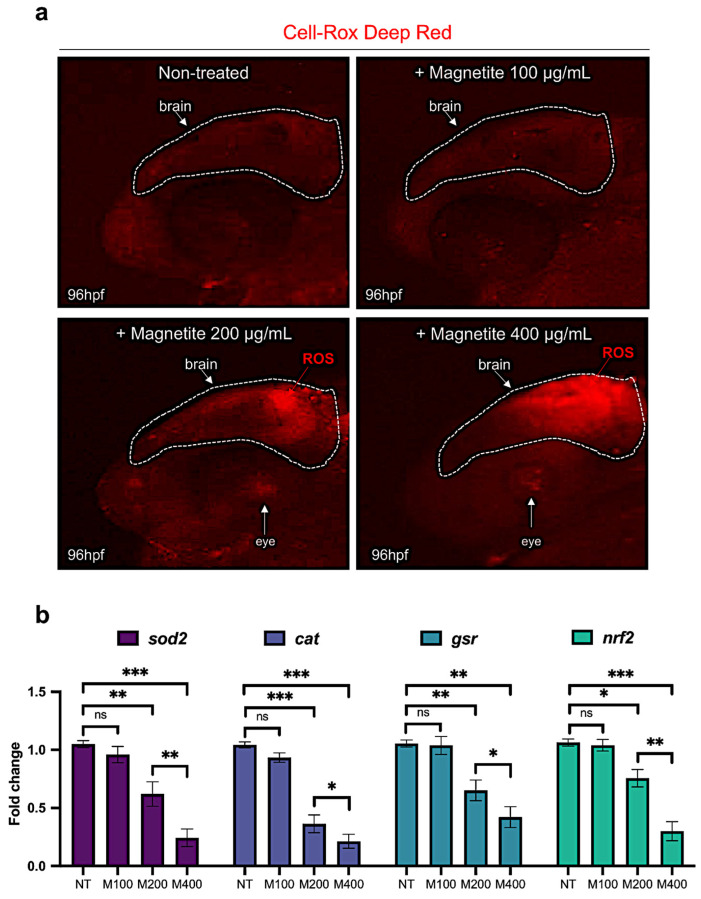
(**a**) Oxidative stress detection (ROS are indicated with red arrow) in brain from embryos treated with different concentrations of magnetite (100; 200; 400 µg/mL) (lateral view of the head); microscope’s magnification 20× (**b**) qRT-PCR gene expression analysis of *sod2*, *cat*, *gsr*, and *nrf2* in dissected heads from control and magnetite-treated (M) embryos at 96 hpf (for each group, *n* = 10 heads were pooled). Each experiment was repeated independently three times. Statistical significance was calculated by one-way ANOVA (multiple comparison Tukey–Kramer post hoc test) (* *p* < 0.01; ** *p* < 0.001; *** *p* < 0.0001; ns = not significant). Center values denote the mean ± SD.

Previous in vitro studies showed that cells treated with magnetite present a significant induction of ROS and increased apoptosis [34]. In detail, the authors showed that magnetite selectively releases Fe cations inside cells which, due to Fenton reactions, promotes the formation of ROS and apoptosis. To verify this hypothesis in our in vivo system, we treated zebrafish embryos with different sub-lethal concentrations of magnetite, and then we detected the level of oxidative stress by the CellROX Deep Red assay. As shown in Figure 5a, we observed a significant increase in ROS levels in the brain from embryos treated with magnetite at concentrations of 200 and 400 µg/mL.

**Figure 6:**
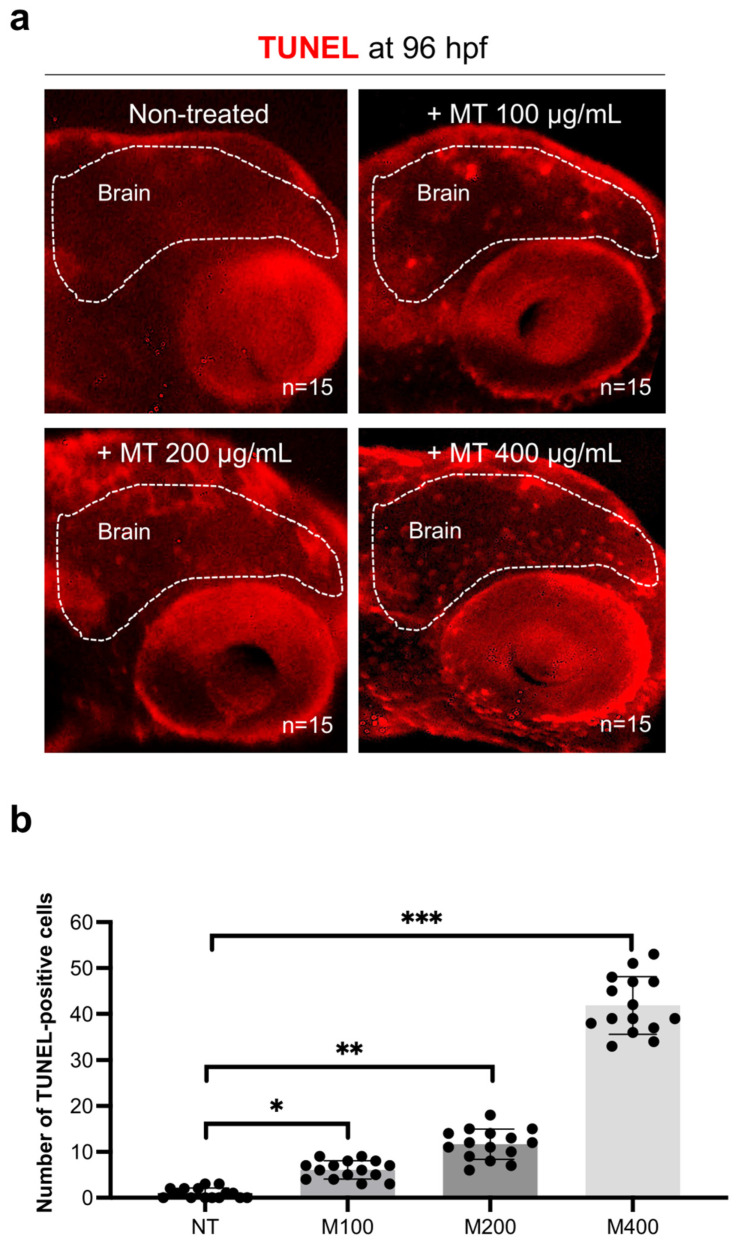
(**a**) TUNEL staining of brain apoptotic cells (see white rectangle) from controls and embryos treated with different concentrations of magnetite (100; 200; 400 µg/mL) at 96 hpf (lateral view of the head); microscope’s magnification 20×; (**b**) counting of TUNEL-positive cell number. Each experiment was repeated independently three times (*n* = 15 animals for each condition). (M = magnetite) Statistical significance was calculated by one-way ANOVA (multiple comparison Tukey–Kramer post hoc test) (* *p* < 0.01; ** *p* < 0.001; *** *p* < 0.0001). Center values denote the mean ± SD.

Differently, no significant levels were detected in embryos treated with 100 ug/mL magnetite or controls (Figure 5a). Consistently, zebrafish embryos treated with magnetite displayed a significant decrease in brain expression levels of key antioxidant genes: superoxide dismutase (*sod2*), catalase (*cat*), and glutathione reductase (*gsr*) and the transcription factor (*nrf2*) (Figure 5b). Next, we also evaluated the apoptosis at 96 hpf by using the TUNEL assay kit. We found a significant dose-dependent increase in the number of TUNEL-positive cells in the brain from magnetite-treated embryos than un-treated controls (Figure 6a,b).

### 2.6. Magnetite Treatment Decreases Neural Progenitors in Zebrafish Embryo

**Figure 7:**
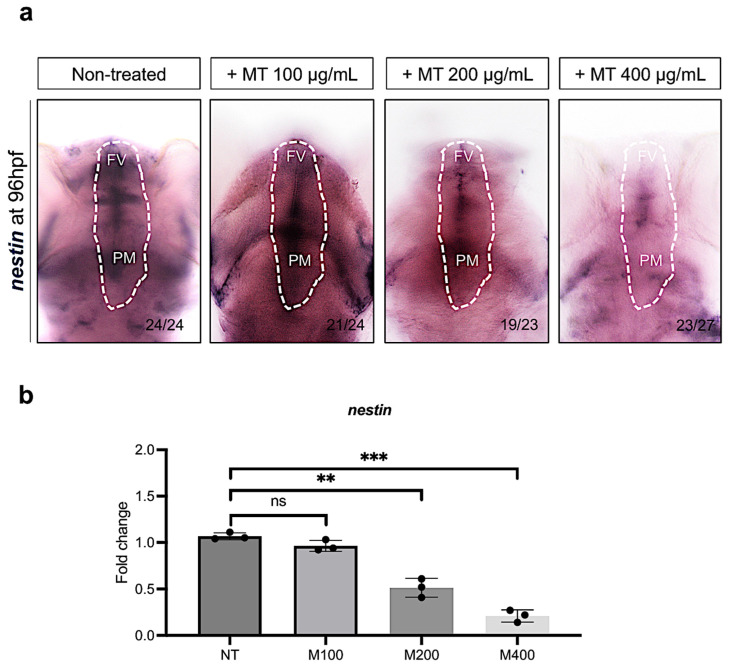
(**a**) WISH for *nestin* in controls and magnetite-treated (MT) embryos (100, 200, and 400 µg/mL) at 96 hpf (dorsal view). White line highlights two specific regions: FV (ventricular forebrain) and PM (post-midbrain); microscope’s magnification 20× (**b**) qRT-PCR data examining *nestin* expression levels (fold change relative to expression in un-treated embryos) in dissected heads from embryos at 96 hpf (for each group, *n* = 30 heads were pooled). Each experiment was repeated independently three times. Statistical significance was calculated by one-way ANOVA (multiple comparison Tukey–Kramer post hoc test) (** *p* < 0.001; *** *p* < 0.0001; ns = not significant). Center values denote the mean ± SD.

To evaluate the impact of magnetite during embryonic neurogenesis, as mentioned before, we treated embryos with magnetite (MT) (100, 200, and 400 µg/mL) and then fixed them at 96 hpf. To evaluate the effects of magnetite on neural progenitor cells, we analyzed the expression levels of the *nestin* gene, described as the main marker of these precursors [35]. Embryos treated with magnetite showed a significant dose-dependent reduction in *nestin*-expressing cells in the ventricular forebrain (FV) and the post-midbrain (PM) compared to non-treated embryos (Figure 7a). Consistently, the qRT-PCR analysis showed that total brain *nestin* transcript levels were lower in the magnetite-treated (200 and 400 µg/mL) group than in the un-treated group (Figure 7b).

**Figure 8:**
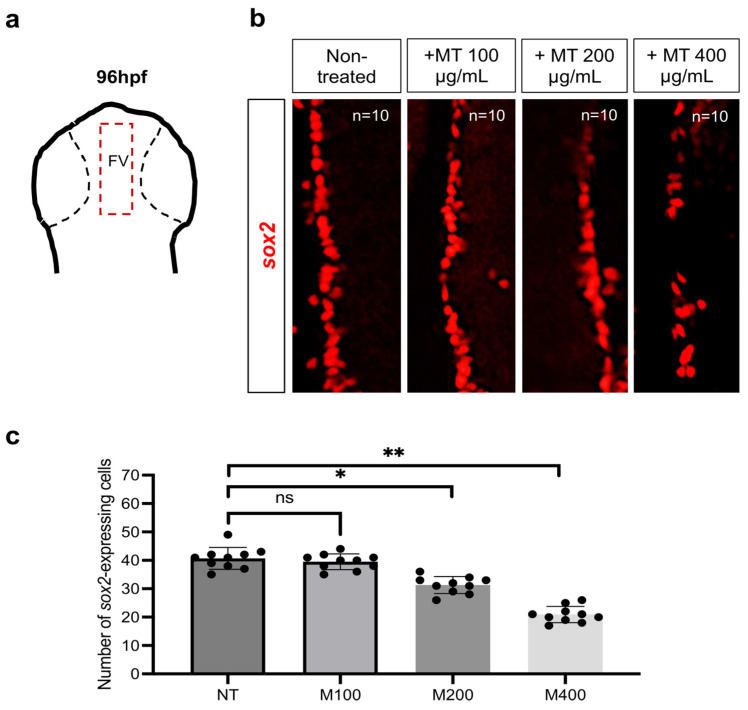
(**a**) Schematic region (dorsal view) of the ventricular forebrain (FV). (**b**) Fluorescence in situ hybridization for *sox2* in FV of control and treated embryos (100, 200, and 400 µg/mL of magnetite MT) at 96 hpf (dorsal view). Each experiment was repeated independently three times. (**c**) Statistical significance was calculated by one-way ANOVA (multiple comparison Tukey–Kramer post hoc test) (* *p* < 0.01; ** *p* < 0.001; ns = not significant). Center values denote the mean ± SD.

In parallel, we also evaluated the expression levels of the transcription factor *sox2* (marker of stem cell progenitor) by using fluorescence in situ hybridization. We found a significant lowering of the *sox2*-expressing cell number in the ventricular forebrain region of magnetite-treated embryos compared to controls (Figure 8a–c).

### 2.7. Magnetite Treatment Affects Neural Cell Proliferation in Embryo Zebrafish Brain

**Figure 9:**
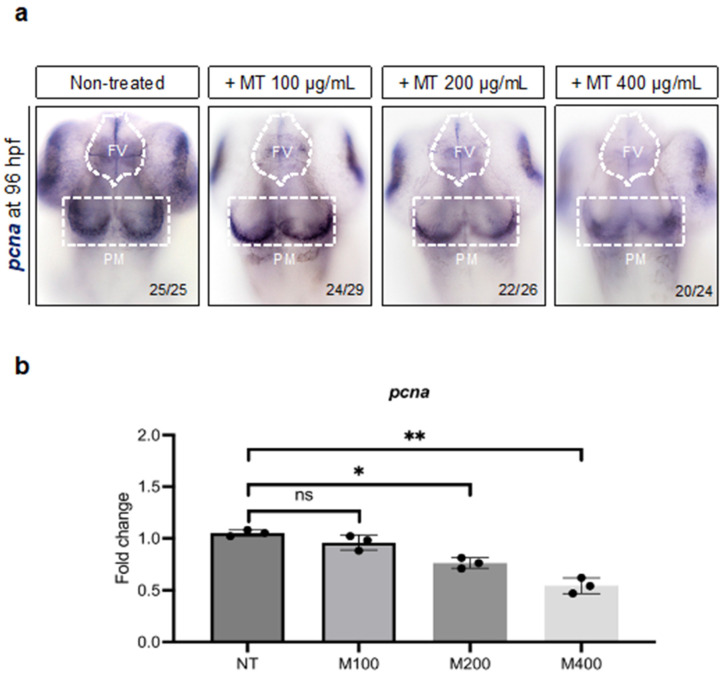
(**a**) WISH for *pcna* in magnetite (MT)-treated and non-treated embryos at 96 hpf (dorsal view). White line highlights FV (ventricular forebrain) and PM (post-midbrain) regions; microscope’s magnification 20×; (**b**) qRT-PCR data examining *pcna* expression (fold change relative to expression in non-treated embryos) in dissected heads from embryos at 96 hpf (for each group, *n* = 30 heads were pooled). Each experiment was repeated independently three times. Statistical significance was calculated by one-way ANOVA (multiple comparison Tukey–Kramer post hoc test) (* *p* < 0.01; ** *p* < 0.001; ns = not significant). Center values denote the mean ± SD.

To evaluate whether the proliferative potential of neural precursors could be affected by magnetite treatment, we analyzed the expression of the proliferating cell nuclear antigen gene (*pcna*), a marker of cell proliferation, which is highly expressed during neurogenesis [36]. As shown, we found a significative reduction in *pcna*-expressing cells in the FV and PM brain regions in treated embryos (200 and 400 µg/mL) compared to non-treated ones (Figure 9a). Consistently, a significant reduction in the total brain *pcna* transcript was found in treated embryos, a result found via the qRT-PCR analysis (Figure 9b).

### 2.8. Magnetite Exposure Increases Inflammation and Microglia Cells in Embryo Zebrafish Brain

**Figure 10:**
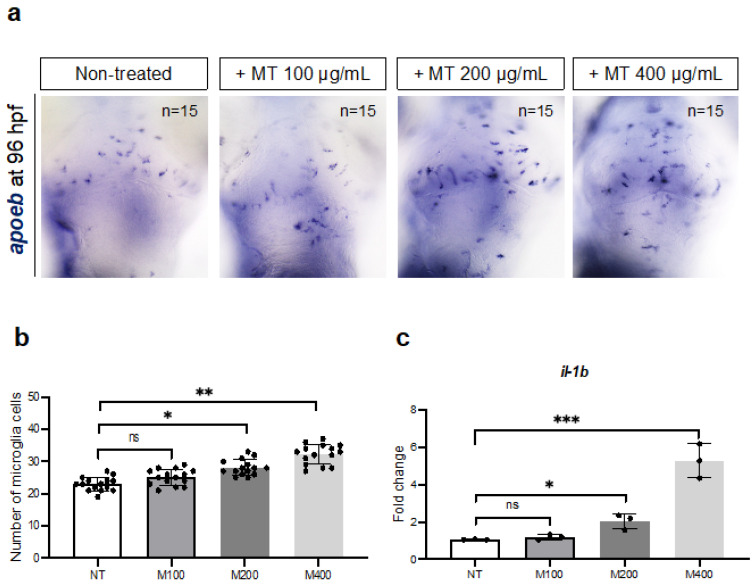
(**a**) WISH for *apoeb* (microglia marker) in non-treated and treated embryos at 96 hpf (dorsal view of the brain), microscope’s magnification 20×; (**b**) Counting of *apoeb*-expressing cells. Statistical significance was calculated by one-way ANOVA (multiple comparison Tukey–Kramer post hoc test) (* *p* < 0.01; ** *p* < 0.001; ns = not significant). (**c**) qRT-PCR data examining *il1b* expression (fold change relative to expression in non-treated embryos) in dissected heads from embryos at 96 hpf (for each group, *n* = 30 heads were pooled). Statistical significance was calculated by one-way ANOVA (multiple comparison Tukey–Kramer post hoc test) (* *p* < 0.01; *** *p* < 0.0001; ns = not significant). Each experiment was repeated independently three times. Center values denote the mean ± SD.

Based on our results, we found that magnetite increased oxidative stress and apoptosis; thus, we also investigated the impact of magnetite on microglia cells. These cells represent a specific macrophage population resident in the brain, which plays a crucial role during embryonic development. We performed in situ hybridization for the *apoeb* gene (specific maker of zebrafish microglial cells) in dissected heads from control and magnetite-treated embryos (Figure 10a,b). We observed a significant boost in the microglial cell number in the brains from treated embryos. In addition, by qRT-PCR analysis, we also found an increase of the interleukin-1b (*il1b*) gene, described as the main mediator of inflammation (Figure 10c). These data strongly support the hypothesis that magnetite induces brain inflammation during embryonic development.

**Figure 11:**
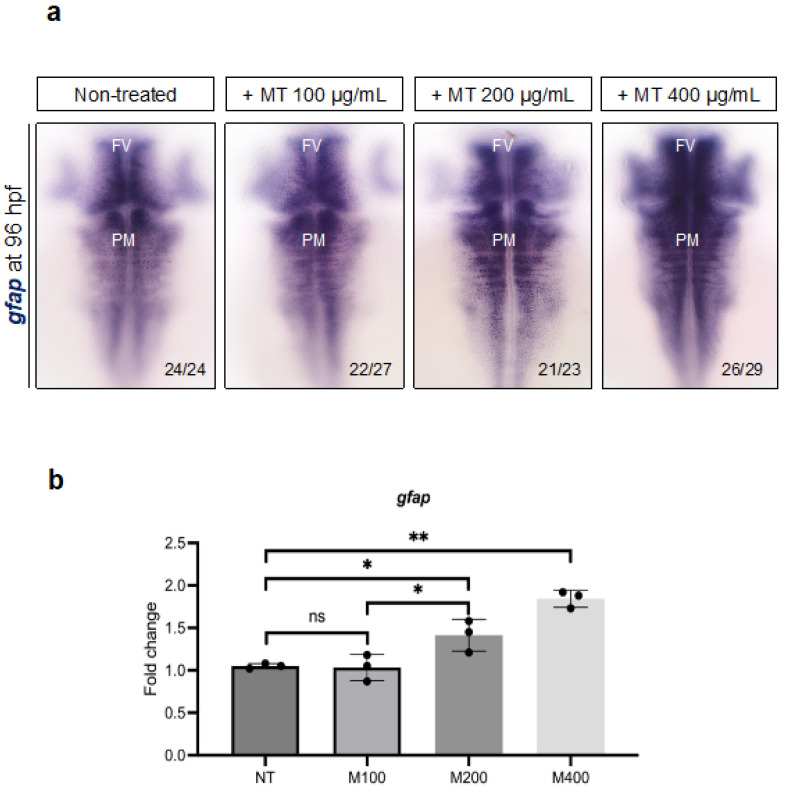
(**a**) WISH for *gfap* in (dorsal view) non-treated and MT-treated embryos at 96 hpf. FV (ventricular forebrain); PM (post-midbrain) microscope’s magnification 20×; (**b**) qPCR data examining *gfap* expression (fold change relative to expression in non-treated embryos) in dissected heads from embryos at 96 hpf (for each group, *n* = 30 heads were pooled). Each experiment was repeated independently three times. Statistical significance was calculated by one-way ANOVA (multiple comparison Tukey–Kramer post hoc test) (* *p* < 0.01; ** *p* < 0.001; ns = not significant). Center values denote the mean ± SD.

Next, we verified the effects of magnetite exposure on the glial cell population by measuring the expression of the glial fibrillary acidic protein (*gfap*), a main marker [37]. As shown in Figure 11, we found a significative increase in *gfap* in the ventricular zone of the forebrain and posterior midbrain (Figure 11a) in embryos treated with magnetite (200 and 400 µg/mL) compared to un-treated embryos. We confirmed this observation by a qRT-PCR analysis of *gfap* transcript levels in the total brain. The results showed a significant increase in this marker in embryos exposed to magnetite (200 and 400 µg/mL) (Figure 11b).

### 2.9. Impact of Magnetite Exposure on Embryo Neuron Specification

**Figure 12:**
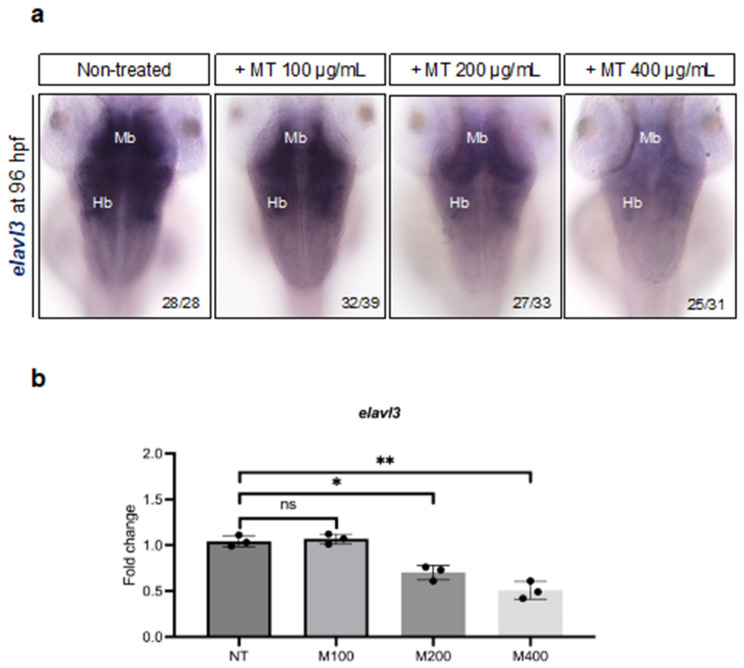
(**a**) WISH for *elavl3* gene in un-treated and MT-treated embryos at 96 hpf (dorsal view). Mb (midbrain); Hb (hindbrain) microscope’s magnification 20×;(**b**) qPCR data examining *elavl3* gene expression (fold change relative to expression in un-treated embryos) in dissected heads from embryos at 96 hpf (for each group, *n* = 30 heads were pooled). Each experiment was repeated independently three times. Statistical significance was calculated by one-way ANOVA (multiple comparison Tukey–Kramer post hoc test) (* *p* < 0.01; ** *p* < 0.001; ns = not significant). Center values denote the mean ± SD.

Lastly, we tested the effect of magnetite on neuronal cells. Thus, we examined the expression of the *elavl3* gene, also known as *huc*, the earliest marker of pan-neuronal cells in zebrafish embryos [38]. Embryos treated with magnetite (200 and 400 µg/mL) presented a significant decrease in *elavl3*-expressing cells in the midbrain and hindbrain regions compared to controls (Figure 12a). This evidence was also confirmed by a total brain *elavl3* gene expression evaluation (Figure 12b).

## 3. Discussion

In the present study, we investigated the effects of magnetite on embryonic neurogenesis using one of the most suitable in vivo models in neurobiology research: the zebrafish model. Firstly, we confirmed the mineral phase of the starting commercial magnetite powder by defining the particles’ morphometry and the dimensional distribution by PXRD and SEM-EDXS analyses. Additionally, to verify the presence of a nanosized component in the particle population, we performed an ARM analysis that highlighted the presence of a small portion of nanosized magnetite agglomerates in the sample that would have been administered to zebrafish embryos. Subsequently, in agreement with previous studies [27,39], we established that the exposure of fish embryos to magnetite concentrations up to 400 µg/mL does not affect embryo viability, as it does not induce morphological defects in key tissues/organs. With the great advantage of embryo transparency presented by the zebrafish model, we were able to measure in vivo the redox state within the brain of magnetite-treated fishes, confirming a dose-dependent increase in ROS levels. The increase in ROS levels might be associated with magnetite coarse particles, with the small portion of nanoparticles, or with the nanosized cleavage fragments following different pathways: (i) the particles or cleavage fragments are acting as Fe reservoirs, but are not directly participating in the ROS generation; (ii) the generation of ROS is promoted at the surface of the particles or of the cleavage fragments by surface-bound Fe; (iii) mineral surface defects can react with the surrounding media to generate ROS [40]. The detection of an average valence state of 2.48 (σ*_n_*_−1_ = 0.11) on the nanosized component of our sample confirmed the possibility of a Fenton reaction to happen, generating ROS. Consistently, after magnetite exposure, brain antioxidant genes (including the transcription factor *nrf2*) were observed to be decreased, while the apoptotic cells increased significantly. Our data reinforced the results obtained by other research groups on in vitro cell models, showing that magnetite treatment promotes oxidative stress and cell death [31]. During embryonic life in humans, maternal exposure to air pollutants was observed to affect neurodevelopment as well as brain postnatal maturation [41,42]. Several human studies described a positive correlation between the accumulation in the brain of elemental Fe, magnetic Fe oxides (including magnetite), and magnetite nanosized cleavage fragments and the development of neurodegenerative features and cognitive dysfunction [43,44,45]. However, to date, no previous study has investigated whether brain magnetite accumulation might have an impact on neurogenesis events. Here, we evaluated key cell markers of embryonic neurogenesis. Specifically, developmental neurogenesis in zebrafish relies on stem cells producing zones, such as in midbrain and forebrain, that contain neuronal precursors with distinct fates. Within these areas, after magnetite treatment, we observed a significant reduction in multiple neurogenesis genes, described as key markers of neural and proliferative progenitors. A summary of the marker genes analyzed is shown in Table 1.

The reduced expression of neurogenesis markers in fish embryos exposed to magnetite let us also hypothesize a potential impairment in the downstream neuronal and glial progenies. Magnetite-exposed embryos, indeed, showed lower *elavl3*-expressing cell number, identifiable as “new born” neurons, while *apoeb* (microglia cell marker), *il1b*, and *gfap* (glial cell marker) increased, confirming a status of neuroinflammation.

## 4. Material and Methods

### 4.1. Powder X-ray Diffraction (PXRD)

Commercial magnetite particles (Inoxia, Ltd., Cranleigh, UK, no. 0029882792582) were first characterized by PXRD. The PXRD data were collected on the sample using a Miniflex 600 diffractometer (Rigaku, Tokyo, Japan) equipped with a Cu–Kα1 radiation source (λ = 1.54055 Å, 40 mA, 45 kV), fixed divergence slits, and a multistrip D/TEX Ultra detector with a resolution of <200 eV. A divergent slit width of 2 mm and a scatter-slit width of 4 mm were used for the incoming beam, whereas a receiving slit width of 0.5 mm and scatter-slit width of 0.2 mm were used for the diffracted beam. Data were collected in step-scan mode in the 3°–70° 2θ range, with a step size of 0.02° 2θ and a counting time of 2 s per step. Samples bearing metals have problems of background. To eliminate the elevated background and fluorescence phenomenon, the detector multistrip D/tex was added with the function “XRF Reduction”. The sample was put in a holder made of Al using the “side loading” technique to avoid a preferred orientation of the particles [46].

### 4.2. Scanning Electron Microscopy

The SEM investigation was performed using a SEM (Tescan Vega 3^®^, Brno, Czech Republic) operating at high vacuum and at a voltage of 30 KeV. The SEM was equipped with an Energy-Dispersion X-Ray Detector (EDXS) (Oxford, Abingdon, UK) managed by AztecONE software version 6.0 (Oxford, Abingdon, UK). The magnetite particles were briefly suspended in ethanol and transferred onto an Al SEM stub covered by carbon tape (Media System Technologies^®^, Macherio, MB, Italy). Pictures were acquired at 1000× magnification then edited and measured with ImageJ software (https://imagej.net) FIJI distribution, NIH, Bethesda, MD, USA), [47]. After drawing the perimeter of each particle, multiple dimensional parameters were automatically calculated by the software. Among these parameters, we selected the Feret diameter to generate the dimensional distribution of the investigated population using the XLSTAT plugin for Excel 2021 (Addinosoft, NY, USA). Furthermore, we collected several EDXS spectra on different particles to confirm the identity of the material by means of a semi-quantitative standardless analysis. Each spectrum was collected with a live time of 20 s and elaborated using the AztecOne software.

### 4.3. Atomic Resolution Scanning Electron Microscopy (ARM) and Dual-Electron Energy-Loss Spectroscopy (Dual-EELS)

The commercial magnetite sample was suspended in 2-propanol and thoroughly shacked. A droplet of the magnetite sample suspended in 2-propanol was transferred with a pipette on a 200 mesh TEM lacey carbon copper grid (SPI Supplies, West Chester, PA, USA) positioned within inverted tweezers to allow for the collection of all the suspended material. After the transfer, the droplet was left drying before mounting the grid onto the TEM holder. The goal of this analysis was to determine the possible presence of a small amount of magnetite nanoparticles or nanosized cleavage fragments in the coarse magnetite commercial sample. This study allowed to further confirm the identity of the minerals used to treat the zebrafish embryos and determine the average Fe valence state of the magnetite nanoparticles and cleavage fragments. These investigations were performed using an aberration-corrected Scanning/Transmission Electron Microscope (acSTEM), model ARM 200 CF, equipped with a high-brightness cold-field emission gun (CFEG) operating at 80 kV, an energy-dispersive X-ray spectroscopy (EDXS) system (Centurio 100 mm^2^, JEOL, Tokyo, Japan), and an energy filter (Quantum GIF, Gatan—Warrendale, PA, USA). The recording of the Dual-range EELS spectra was performed with a collection semi-angle of 60.39 mrad, with a convergence semi-angle of 24.00 mrad, and using an aperture of 5 mm. The collection of the EELS spectra on the region of interest (ROI) was performed in dual mode with a dispersion of 0.25 eV/channel. Both the low-loss region (used for centering the beam only) and the core-loss regions (located at the Fe L_2,3_-edge) were recorded summing up three frames, allowing for an early screening on the ELNES for the possible presence of artefacts due to the exposition of the sample to the electron beam [48]. Each frame of the low-loss region was recorded over a period of 0.001 s, whereas each frame of the core-loss regions was recorded over a period of 30 s for the Fe L_2,3_-edge. The HQ dark correction was applied to reduce the noise originated when working with summed spectra. The Fe valence state in the selected region of interest (ROI) on the magnetite particle boundaries was determined by using the Fe-L_2,3_ white-line intensity ratio by using the universal curve as already applied in similar experimental conditions to guarantee the repeatability of the results [31].

### 4.4. Zebrafish Embryos

Fishes were raised according to FELASA and European guidelines. No authorization was required since all experiments were performed before 5 days post-fertilization. All efforts were made to comply to the 3R guidelines. Embryos were obtained as described previously [49,50]. Zebrafish embryos were treated from 4 h post-fertilization (hpf) up to 96 hpf with different magnetite concentrations (100; 200; 400; 600; 800 µg/mL) to define the survival rate and morphology defects. The magnetite concentrations were established based on a previous toxicology analysis performed by Jurewicz and colleagues [51]. Only fertilized eggs with a normal developmental phase were used for the experiments. Every experiment was performed in triplicate.

### 4.5. Reactive Oxygen Species (ROS) Detection and TUNEL Assay

The detection of oxidative stress was performed on living zebrafish embryos at 96 hpf by using CellROX Deep Red (Invitrogen, Waltham, MA, USA). Magnetite-treated and control embryos were exposed to 5 µM of the CellROX solution for 30 min at 28 °C, followed by analysis using fluorescence microscopy, as previously described [52]. Apoptotic cells were examined by the TUNEL assay. Embryos were fixed with 2% paraformaldehyde overnight at 4 °C. After gradual rehydration, embryos were permeabilized with 25 µg/mL of proteinase K for 10 min at 28 °C followed by treatment with 4% paraformaldehyde and incubated with 90 µL labeling solution plus 10 µL enzyme solution (In Situ Cell Death TUNEL Detection Kit, Roche Diagnostic, Chicago, IL, USA) at 37 °C for 2 h. Embryos were washed three times with PBT for 5 min, and the images were examined by confocal microscopy.

### 4.6. Whole-Mount In Situ Hybridization (WISH)

The digoxigenin-labeled probes *nestin*, *pcna*, *elavl3*, *gfap*, *sox2*, and *apoeb* were generated as previously described [53]. Whole-mount in situ hybridization was performed on 4% paraformaldehyde-fixed embryos at 96 hpf following the protocol previously described [54]. Embryos were imaged in 100% glycerol using a Compact Multi-Lens Stereo Microscope (AM-Scope) with a Digital Eyepiece Camera (Swift company, Swiss, Bern). For fluorescence revelation, all embryos were immersed in anti-DIG POD antibody (Roche Diagnostic, Chicago, IL, USA) (1:200) in the above-described blocking solution for 24 h at room temperature. Next, embryos were washed 4 times in PBS (5 min each). Afterwards, embryos were visualized using the Red Fluorescent Detection set (Roche Diagnostic, Chicago, IL, USA) according to the kit’s instructions using a Leica (Leica Microsystems, Wetzlar, Germany) SP2 confocal microscope. The synthesis of riboprobes was carried out by using the primer sequences listed in Table 2.

### 4.7. RNA Extraction and Reverse Transcription

To extract the total RNA, 120 embryo heads (30 heads for each condition) at 4 days post-fertilization (dpf) were dissected, pooled, and dissociated by using an RNAeasy minikit (Qiagen, Frankfurt, Germany). To obtain purified RNA, we followed the manufacturer’s protocol. This procedure was repeated in three independent experiments (90 embryos in total for each condition). For reverse transcription into cDNA, 0.5 μg of total RNA was incubated with a buffer mix and enzyme using the Superscript III First-Strand Synthesis System kit (Invitrogen, Boston, MA, USA). In detail, 10 μL of the total volume was incubated for 10 min at 25 °C, 30 min at 50 °C, and 5 min at 85 °C. Next, the samples were treated with RNase-H (ThermoFisher Scientific, Waltham, MA, USA) for 30 min at 37 °C.

### 4.8. Quantitative Real-Time Polymerase Chain Reaction (qRT-PCR)

Quantitative RT-PCR experimental procedures were performed by using a thermocycler with a MyiQ detector (Bio-Rad, Hercules, Dallas, TX, USA). Briefly, we mixed cDNA, specific forward and reverse primers, SYBR-Green (Bio-Rad, Hercules, Dallas, TX, USA), and RNase-free water according to the manufacturer’s protocol. The previous mix was incubated for 15 min at 95 °C, for 15 s at 95 °C for 40 cycles, for 30 s at 60 °C for 40 cycles, and for 30 s at 72 °C for 40 cycles. The primer sequences for the PCR gene amplification are listed in Table 3. Data are represented as the fold change of mRNA levels in magnetite-treated embryos on mRNA levels in un-treated controls using *ef1a* to normalize the absolute quantification, calculated using 2^−∆∆Ct^. To confirm the correct amplification, we performed a melting curve analysis and verified the PCR’s efficiency. Each qRT-PCR experiment was performed using biological triplicates. In the qRT-PCR analyses, each *n* represents the average of biological triplicates from a single experiment. All experiments were repeated at least three times.

### 4.9. Statistical Procedures

Data were analyzed with GraphPad Prism 9.4.1 software (GraphPad Inc., San Diego, CA, USA). Statistical comparisons were performed using a one-way ANOVA Tukey’s multiple comparison test; *p* values equal to or less than 0.05 were considered statistically significant.

## 5. Conclusions

Taken together, our findings represent the first evidence in vivo of the effects of magnetite on developing brain cells during embryogenesis. Despite more extensive studies being needed to clarify whether elemental Fe or magnetite cleavage fragments exert the damaging increase of ROS and promote cell death, this study represents possible insight into the specific mechanism connecting magnetite pollutant exposure and morpho-functional brain defects during development.

## Figures and Tables

**Table 1:** Summary of cell marker analysis.

	Non-Treated	Magnetite 100 µg	Magnetite 200 µg	Magnetite 400 µg
*Nestin*	+++	+++	++	+
*sox2*	+++	+++	++	+
*Pcna*	+++	+++	++	+
*elavl3*	+++	+++	++	+
*Gfap*	+++	+++	++++	+++++
*Apoeb*	+++	+++	++++	+++++
*il1b*	+++	+++	++++	+++++

**Table 2:** Primers sequences for synthesis of WISH probes.

*Nestin*	F: 5′-GCAGCCAACAACTATCAGAAAC-3′	R: 5′-CATCGAGGTACTGCTTGGT-3′
*Pcna*	F: 5′-CCTTAAGAAGGTCCTGGAG-3′	R: 5′-CCACACAACTGTATTCCTGCTC-3′
*elavl3*	F: 5′-CCATGGAAACTCAGGTGTC-3′	R: 5′-GTCAGCTGCTCCTAGT-3′
*gfap*	F: 5′-CATCTATCAGGAGGAGCTG-3′	R: 5′-CTCAGCTGGCGCTCCA-3′
*sox2*	F: 5′-CCCTGATGAAGAAGGACAAGT-3′	R: 5′-GTTGTGCGCGTTCAAACTC-3′
*apoeb*	F: 5′-CACAAACTGACGGCATGGT-3′	R: 5′-CGGTTCTTCACGTCATCTG-3′

**Table 3:** Primer sequences for qPCR gene amplification.

*nestin*	F: 5′-CTTCAACATCTTCAGGCCCAAG-3′	R:5′-GTGTTGGTCTGTCGATTCTCAG-3′
*pcna*	F: 5′-CAAGGAGGATGAAGCGGTAACA-3′	R: 5′-CTGCGGACATGCTAAGTGTG-3′
*elavl3*	F: 5′-GCCAGCTACGGAGTCAAGAG-3′	R: 5′-CATGGTGACGAAGCCAAAGC-3′
*gfap*	F: 5′-ACCCGTGACGGAGAGATCAT-3′	R: 5′-GCCAGTGTCTGAGCCTCATT-3′
*sod2*	F: 5′-CAGCAAGCACCATGCAACAT-3′	R: 5′-CAGCTCACCCTGTGGTTCTC-3′
*cat*	F: 5′-TGAGGCTGGGTCATCAGATA-3′	R: 5′-AAAGACGGAAACAGAAGCGT-3′
*gsr*	F: 5′-CTCCTTGGTCGCAGCATGGCT-3′	R: 5′-GGCAGTGGTGGCACCGAGTTC-3′
*nrf2*	F: 5′-TGTTGGTTCGGAGGCTCTTAA-3′	R: 5′-AGGCCATGTCCACACGTACA-3′
*il1b*	F: 5′-ATGGCGAACGTCATCCAAGA-3′	R: 5′-GAGACCCGCTGATCTCCTTG-3′
*ef1a*	F: 5′-CCTGGGAGTGAAACAGCTG-3′	R: 5′-GCCTCCAGCATGTTGTCAC-3′

## Data Availability

All data generated in this study are available from the corresponding author on reasonable request.

## Supplementary Materials

The supporting information can be downloaded at https://www.mdpi.com/article/10.3390/ijms25126459/s1.

## References

[1] Accogli A., Addour-Boudrahem N., Srour M. (2020). Neurogenesis, neuronal migration, and axon guidance. Handb. Clin. Neurol..

[2] Altbuerger C., Rath M., Armbruster D., Driever W. (2024). Neurog1 and Olig2 integrate patterning and neurogenesis signals in development of zebrafish dopaminergic and glutamatergic dual transmitter neurons. Dev. Biol..

[3] Allende M.L., Weinberg E.S. (1994). The Expression Pattern of 2 Zebrafish Achaete-Scute Homolog (Ash) Genes Is Altered in the Embryonic Brain of the Cyclops Mutant. Dev. Biol..

[4] Spassky N., Aguilar A. (2008). Shh regulates neurogenesis through primary cilia. Med. Sci..

[5] Wang X.K., Emelyanov A., Korzh V., Gong Z.Y. (2003). Zebrafish homologue is expressed during neurogenesis in embryonic development. Dev. Dynam.

[6] Samarut E., Bekri A., Drapeau P. (2016). Transcriptomic Analysis of Purified Embryonic Neural Stem Cells from Zebrafish Embryos Reveals Signaling Pathways Involved in Glycine-Dependent Neurogenesis. Front. Mol. Neurosci..

[7] Valero J., Paris I., Sierra A. (2016). Lifestyle Shapes the Dialogue between Environment, Microglia, and Adult Neurogenesis. ACS Chem. Neurosci..

[8] Briones T.L. (2006). Environment, physical activity, and neurogenesis: Implications for prevention and treatment of Alzhemier’s disease. Curr. Alzheimer Res..

[9] Costa L.G., Cole T.B., Coburn J., Chang Y.C., Dao K., Roque P.J. (2017). Neurotoxicity of traffic-related air pollution. Neurotoxicology.

[10] Boda E., Rigamonti A.E., Bollati V. (2020). Understanding the effects of air pollution on neurogenesis and gliogenesis in the growing and adult brain. Curr. Opin. Pharmacol..

[11] Ward R.J., Zucca F.A., Duyn J.H., Crichton R.R., Zecca L. (2014). The role of iron in brain ageing and neurodegenerative disorders. Lancet Neurol..

[12] Liu X.Q., Huang J., Song C., Zhang T.L., Liu Y.P., Yu L. (2023). Neurodevelopmental toxicity induced by PM2.5 Exposure and its possible role in Neurodegenerative and mental disorders. Hum. Exp. Toxicol..

[13] Waidyatillake N.T., Campbell P.T., Vicendese D., Dharmage S.C., Curto A., Stevenson M. (2021). Particulate Matter and Premature Mortality: A Bayesian Meta-Analysis. Int. J. Environ. Res. Public Health.

[14] Liu C., Chen R., Sera F., Vicedo-Cabrera A.M., Guo Y., Tong S., Coelho M., Saldiva P.H.N., Lavigne E., Matus P. (2019). Ambient Particulate Air Pollution and Daily Mortality in 652 Cities. N. Engl. J. Med..

[15] Fan J.X., Yang J.X., Cheng F.L., Zhang S.K. (2023). The Source, Distribution, and Environmental Effects of Suspended Particulate Matter in the Yangtze River System. Water.

[16] Zhao Z.L., Wang L., Shi W.H., Li C., Wei G.Z.J. (2022). Motion Adsorption Characteristics of Particulate Matter in Water Supply Network. Water.

[17] Collingwood J.F. (2018). Brain iron dysregulation in neurodegenerative disease: Insights from systems modelling and synchrotron spectromicroscopy. Febs. Open Biol..

[18] Pankhurst Q., Hautot D., Khan N., Dobson J. (2008). Increased levels of magnetic iron compounds in Alzheimer’s disease. J. Alzheimers Dis..

[19] Giere R. (2016). Magnetite in the human body: Biogenic vs. anthropogenic. Proc. Natl. Acad. Sci. USA.

[20] Tabner B.J., Mayes J., Allsop D. (2010). Hypothesis: Soluble abeta oligomers in association with redox-active metal ions are the optimal generators of reactive oxygen species in Alzheimer’s disease. Int. J. Alzheimers Dis..

[21] Maher B.A., Ahmed I.A., Karloukovski V., MacLaren D.A., Foulds P.G., Allsop D., Mann D.M., Torres-Jardon R., Calderon-Garciduenas L. (2016). Magnetite pollution nanoparticles in the human brain. Proc. Natl. Acad. Sci. USA.

[22] Hautot D., Pankhurst Q.A., Khan N., Dobson J. (2003). Preliminary evaluation of nanoscale biogenic magnetite in Alzheimer’s disease brain tissue. Proc. Biol. Sci..

[23] Hussain R., Graham U., Elder A., Nedergaard M. (2023). Air pollution, glymphatic impairment, and Alzheimer’s disease. Trends Neurosci..

[24] Cole-Hunter T., Zhang J., So R., Samoli E., Liu S., Chen J., Strak M., Wolf K., Weinmayr G., Rodopolou S. (2023). Long-term air pollution exposure and Parkinson’s disease mortality in a large pooled European cohort: An ELAPSE study. Environ. Int..

[25] Liu N.M., Miyashita L., Maher B.A., McPhail G., Jones C.J.P., Barratt B., Thangaratinam S., Karloukovski V., Ahmed I.A., Aslam Z. (2021). Evidence for the presence of air pollution nanoparticles in placental tissue cells. Sci. Total Environ..

[26] Coccini T., Pignatti P., Spinillo A., De Simone U. (2020). Developmental Neurotoxicity Screening for Nanoparticles Using Neuron-Like Cells of Human Umbilical Cord Mesenchymal Stem Cells: Example with Magnetite Nanoparticles. Nanomaterials.

[27] Basaki M., Keykavusi K., Sahraiy N., Ali Shahbazfar A. (2021). Maternal exposure to iron oxide nanoparticles is associated with ferroptosis in the brain: A chicken embryo model analysis. J. Anim. Physiol. Anim. Nutr..

[28] Semeano A.T., Tofoli F.A., Correa-Velloso J.C., de Jesus Santos A.P., Oliveira-Giacomelli A., Cardoso R.R., Pessoa M.A., da Rocha E.L., Ribeiro G., Ferrari M.F.R. (2022). Effects of Magnetite Nanoparticles and Static Magnetic Field on Neural Differentiation of Pluripotent Stem Cells. Stem Cell Rev. Rep..

[29] Engert F., Wilson S.W. (2012). Zebrafish neurobiology: From development to circuit function and behaviour. Dev. Neurobiol..

[30] Eisen J.S. (1991). Developmental neurobiology of the zebrafish. J. Neurosci..

[31] Adams M.M., Kafaligonul H. (2018). Zebrafish-A Model Organism for Studying the Neurobiological Mechanisms Underlying Cognitive Brain Aging and Use of Potential Interventions. Front. Cell Dev. Biol..

[32] Schmidt R., Strahle U., Scholpp S. (2013). Neurogenesis in zebrafish—From embryo to adult. Neural Dev..

[33] Grandel H., Kaslin J., Ganz J., Wenzel I., Brand M. (2006). Neural stem cells and neurogenesis in the adult zebrafish brain: Origin, proliferation dynamics, migration and cell fate. Dev. Biol..

[34] Ramesh V., Ravichandran P., Copeland C.L., Gopikrishnan R., Biradar S., Goornavar V., Ramesh G.T., Hall J.C. (2012). Magnetite induces oxidative stress and apoptosis in lung epithelial cells. Mol. Cell Biochem..

[35] Schwarzer S., Asokan N., Bludau O., Chae J., Kuscha V., Kaslin J., Hans S. (2020). Neurogenesis in the inner ear: The zebrafish statoacoustic ganglion provides new neurons from a Neurod/Nestin-positive progenitor pool well into adulthood. Development.

[36] Wullimann M.F., Mueller T. (2004). Identification and morphogenesis of the eminentia thalami in the zebrafish. J. Comp. Neurol..

[37] Nielsen A.L., Jorgensen A.L. (2003). Structural and functional characterization of the zebrafish gene for glial fibrillary acidic protein, GFAP. Gene.

[38] Cacialli P., Ricci S., Frabetti F., Ferrando S., Franceschini V. (2024). Exposure of Zebrafish Embryos to Urea Affects Gene Expression in Neuronal Cells. Environments.

[39] Rocha J.M.V., de Souza V.B., Panunto P.C., Nicolosi J.S., da Silva E.D.N., Cadore S., Londono O.M., Muraca D., Tancredi P., de Brot M. (2022). In vitro and in vivo acute toxicity of a novel citrate-coated magnetite nanoparticle. PLoS ONE.

[40] Schoonen M.A.A., Cohn C.A., Roemer E., Laffers R., Simon S.R., O’Riordan T. (2006). Mineral-induced formation of reactive oxygen species. Rev. Miner. Geochem..

[41] Niklison-Chirou M.V., Agostini M., Amelio I., Melino G. (2020). Regulation of Adult Neurogenesis in Mammalian Brain. Int. J. Mol. Sci..

[42] Bolton J.L., Marinero S., Hassanzadeh T., Natesan D., Le D., Belliveau C., Mason S.N., Auten R.L., Bilbo S.D. (2017). Gestational Exposure to Air Pollution Alters Cortical Volume, Microglial Morphology, and Microglia-Neuron Interactions in a Sex-Specific Manner. Front. Synaptic Neurosci..

[43] Gonet T., Maher B.A., Kukutschova J. (2021). Source apportionment of magnetite particles in roadside airborne particulate matter. Sci. Total Environ..

[44] Maher B.A. (2019). Airborne Magnetite- and Iron-Rich Pollution Nanoparticles: Potential Neurotoxicants and Environmental Risk Factors for Neurodegenerative Disease, Including Alzheimer’s Disease. J. Alzheimers Dis..

[45] Vigliaturo R., Jamnik M., Drazic G., Podobnik M., Znidaric M.T., Ventura G.D., Redhammer G.J., Znidarsic N., Caserman S., Giere R. (2022). Nanoscale transformations of amphiboles within human alveolar epithelial cells. Sci. Rep..

[46] Moore D.M., Reynolds R.C. (1997). X-ray Diffraction and the Identification and Analysis of Clay Minerals.

[47] Schindelin J., Arganda-Carreras I., Frise E., Kaynig V., Longair M., Pietzsch T., Preibisch S., Rueden C., Saalfeld S., Schmid B. (2012). Fiji: An open-source platform for biological-image analysis. Nat. Methods.

[48] Vigliaturo R., Pollastri S., Gieré R., Gualtieri A.F., Drazic G. (2019). Experimental quantification of the Fe-valence state at amosite-asbestos boundaries using acSTEM dual-electron energy-loss spectroscopy. Am. Mineral..

[49] Cacialli P., Dogan S., Linnerz T., Pasche C., Bertrand J.Y. (2023). Minichromosome maintenance protein 10 (mcm10) regulates hematopoietic stem cell emergence in the zebrafish embryo. Stem Cell Rep..

[50] Cacialli P., Mailhe M.P., Wagner I., Merkler D., Golub R., Bertrand J.Y. (2022). Synergistic prostaglandin E synthesis by myeloid and endothelial cells promotes fetal hematopoietic stem cell expansion in vertebrates. EMBO J..

[51] Jurewicz A., Ilyas S., Uppal J.K., Ivandic I., Korsching S., Mathur S. (2020). Evaluation of Magnetite Nanoparticle-Based Toxicity on Embryo-Larvae Stages of Zebrafish (*Danio rerio*). ACS Appl. Nano Mater..

[52] Cacialli P., Mahony C.B., Petzold T., Bordignon P., Rougemont A.L., Bertrand J.Y. (2021). A connexin/ifi30 pathway bridges HSCs with their niche to dampen oxidative stress. Nat. Commun..

[53] Mahony C.B., Cacialli P., Pasche C., Monteiro R., Savvides S.N., Bertrand J.Y. (2021). Hapln1b, a central organizer of the ECM, modulates kit signaling to control developmental hematopoiesis in zebrafish. Blood Adv..

[54] Cacialli P. (2022). Expression of Nerve Growth Factor and Its Receptor TrkA in the Reproductive System of Adult Zebrafish. Vet. Sci..

