# Supplementary material for: Altered Morpho-Functional Features of Neurogenesis in Zebrafish Embryos Exposed to Non-Combustion-Derived Magnetite
Source: Int J Mol Sci. 2024 Jun 12;25(12):6459. doi: 10.3390/ijms25126459 (PMC11203806; doi:10.3390/ijms25126459)
Supplement: Supplementary file 1 [file ijms-25-06459-s001.zip › ijms-3034388-supplementary.pdf]

Supplementary Figure S1.

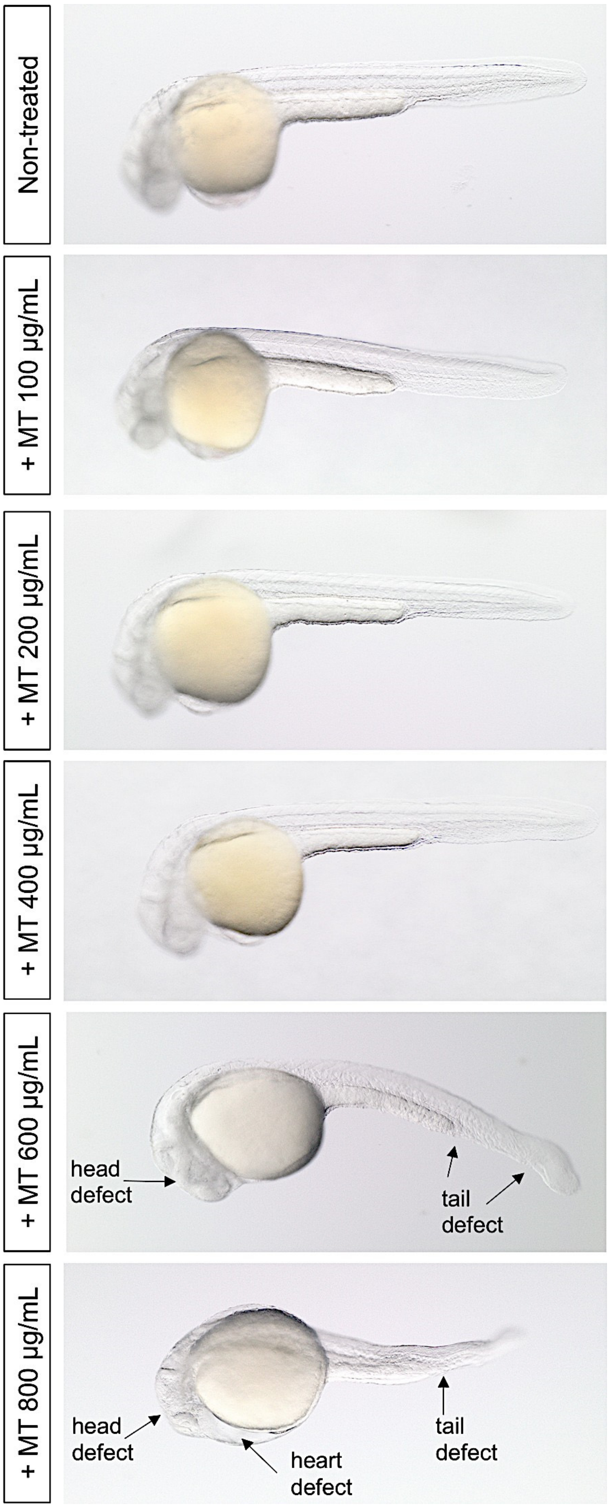

Figure S1. Morphological analysis of zebrafish embryo non-treated and treated with magnetite (MT). Embryos treated with magnetite at 600 and 800 µg/mL present head,heart and tail defects at 24 hours post fertilization.
